# Inverse relationship between serum adenosine deaminase levels and islet beta cell function in patients with type 2 diabetes

**DOI:** 10.1186/s13098-021-00671-2

**Published:** 2021-05-17

**Authors:** Jie Cao, Hong Wang, Jian-bin Su, Xue-qin Wang, Dong-mei Zhang, Xiao-hua Wang, Wang-shu Liu, Xiao-qin Ge

**Affiliations:** 1 Department of Endocrinology, Affiliated Hospital 2 of Nantong University, and First People’s Hospital of Nantong City, No. 6, Haierxiang North Road, Nantong, 226001 China; 2 Medical Research Center, Affiliated Hospital 2 of Nantong University, and First People’s Hospital of Nantong City, No. 6, Haierxiang North Road, Nantong, 226001 China

**Keywords:** Adenosine deaminase, Islet beta cell function, Type 2 diabetes

## Abstract

**Objective:**

Type 2 diabetes (T2D) is a chronic low-grade inflammatory disease, which characterized by islet beta cell dysfunction. Serum adenosine deaminase (ADA) is an important enzyme that regulates the biological activity of insulin, and its levels are greatly increased in inflammatory diseases with insulin resistance. The present study was designed to explore the relationship between serum ADA levels and islet beta cell function in patients with T2D.

**Methods:**

This cross-sectional study recruited 1573 patients with T2D from the Endocrinology Department of the Affiliated Hospital 2 of Nantong University between 2015 and 2018. All participants were received serum ADA test and oral glucose tolerance test (OGTT). Insulin sensitivity index (assessed by Matsuda index using C-peptide, ISI_M-cp_), insulin secretion index (assessed by ratio of area under the C-peptide curve to glucose curve, AUC_cp/glu_) and islet beta cell function (assessed by insulin secretion-sensitivity index 2 using C-peptide, ISSI2_cp_) were derived from OGTT. And other clinical parameters, such as HbA1c, were also collected.

**Results:**

It was showed that HbA1c was significantly increased, while ISI_M-cp_, AUC_cp/glu_ and ISSI2_cp_ significantly decreased, across ascending quartiles of serum ADA levels. Moreover, serum ADA levels were negatively correlated with ISSI2_cp_ (*r* = − 0.267, *p* < 0.001). Furthermore, after adjusting for other clinical parameters by multiple linear regression analysis, serum ADA levels were still independently associated with ISSI2_cp_ (*β* =  − 0.125, *t* =  − 5.397, *p* < 0.001, adjusted *R*^2^ = 0.459).

**Conclusions:**

Serum ADA levels are independently associated with islet beta cell function in patients with T2D.

## Introduction

Adenosine deaminase (ADA) is a key enzyme in purine metabolism that catalyzes the irreversible conversion of adenosine and deoxyadenosine to inosine and deoxyinosine, respectively [1, 2]. ADA is extensively expressed in various human tissues, with the highest activity in thymus, spleen and other lymphoid tissues, and is related to cell mediated immunity [3, 4]. ADA is an important enzyme that regulates the biological activity of insulin [5, 6]. In addition, increasing evidence highlights that adenosine affects insulin secretion and insulin sensitivity, and also plays an important role in regulating islet cell function [7, 8]. Serum ADA activity is strongly increased in autoimmune diseases, cancers and inflammatory diseases [4, 9]. Studies have shown that expression and activity of ADA are directly related to the degree of inflammation [1, 5].

Type 2 diabetes (T2D) is a complex disease caused by polygenic inheritance and environmental interactions, which characterized by chronic low-grade inflammation and hyperglycemia [10–13]. It is well known that insulin resistance and islet beta cell dysfunction are the pathophysiological mechanisms of T2D [14, 15]. Recent studies showed that serum ADA levels and its isoenzymes are significantly higher in patients with T2D than in healthy controls [1, 5]. In addition, serum ADA levels were positively correlated with fasting plasma glucose (FPG), postprandial plasma glucose (PPG) and glycated hemoglobin (HbA1c) in patients with T2D [1, 5, 16]. However, a previous study by Khemka et al. [17] showed that there was no correlation between serum ADA levels and HbA1c in patients with T2D. Moreover, the relationship between serum ADA levels and islet beta cell function in T2D has not been fully elucidated.

Hyperglycemic and euglycemic clamp techniques are internationally recognized as “gold standard” for evaluating insulin secretion and insulin sensitivity, respectively [18]. However, these techniques are so expensive and time-consuming that cannot be widely carried out in clinical practice and large-scale epidemiological studies. Surrogate measures of insulin sensitivity and insulin secretion can be calculated from the results of oral glucose tolerance test (OGTT). Matsuda index (ISI_M-cp_) was used to evaluate the sensitivity of liver and peripheral tissues to insulin, and the ratio of total area under the C-peptide curve to area under the glucose curve (AUC_cp/glu_) was used to evaluate the insulin secretion [19, 20]. Furthermore, the insulin secretion-sensitivity index 2 using C-peptide (ISSI2_cp_), which was the product of ISI_M-cp_ and AUC_cp/glu_, was an integrated indicator of islet beta cell function based on OGTT [21, 22].

Therefore, the present cross-sectional study was designed to investigate the relationship between serum ADA levels and islet beta cell function in patients with T2D.

## Methods

### Study population

This cross-sectional study recruited 1573 individuals, including 890 men and 683 women, who were admitted to the Endocrinology Department of the Affiliated Hospital 2 of Nantong University between 2015 and 2018. The inclusion criteria were (1) diagnosis of T2D according to the statement by the American Diabetes Association in 2011 [23] and (2) serum ADA levels in the normal range of 5-25U/L. The exclusion criteria were as follows: (1) type 1 diabetes; (2) acute complications of diabetes; (3) fasting C-peptide < 0.5 ng/ml; (4) excessive drinking, with alcohol intake > 30 g daily for men and > 20 g daily for women; (5) pregnancy; (6) chronic kidney disease stages 4 and 5; (7) chronic liver disease; (8) abnormal thyroid function; (9) malignant tumors; (10) connective tissue disease; (11) tuberculosis. The study was approved by the ethics committee of the Second Affiliated Hospital of Nantong University and conformed to the Helsinki Declaration.

### Basic data collection

Since all recruited patients were inpatients, the clinical information, physical examination, biochemical data and imaging data could be obtained from the hospital information platform. The main medical history included age, sex, height, weight, systolic blood pressure (SBP), diastolic blood pressure (DBP), diabetes duration, past illness (i.e., hypertension, malignant tumors, thyroid dysfunction, rheumatic diseases, etc.), current medications taken (i.e., statins and antihypertensives) and glucose-lowering therapies (i.e., lifestyle alone, insulin secretagogues, metformin, pioglitazone, glucosidase inhibitors, DPP-4Is, insulin treatments, etc.). Body mass index (BMI) was calculated as weight/the height squared.

### Laboratory data collection

After an overnight fasting, venous blood samples were collected from all recruited patients for biochemical indices. The FPG, triglyceride (TG), total cholesterol (TC), high-density lipoprotein cholesterol (HDLC), low-density lipoprotein cholesterol (LDLC), alanine aminotransferase (ALT) and aspartate aminotransferase (AST) were measured with an automated biochemical analyzer (Model 7600, Hitachi). Serum C-peptide levels were measured with the electrochemiluminescence immunoassays in an immunoassay system (DxI 800, Beckman Coulter). The intra and inter-assay variation coefficients of C-peptide were 2.0–2.8% and 2.3–3.5%, respectively. HbA1c was measured with the ion exchange-based HPLC method in a hemoglobin analysis system (D-10 Testing Program, Bio-Rad). Serum ADA levels were measured by the adenosine deaminase reagent kit (MedicalSystem Biotechnology Company Limited, Ningbo, China) using an automated biochemical analyzer (Model 7600, Hitachi).

### OGTT procedures and islet beta cell function

A standard 75-g oral glucose tolerance test (OGTT) was performed after at least 8 h fast in the morning, and venous blood samples were collected at fasting (0) and at 30, 60, 120 and 180 min after the glucose load to detect serum glucose and C-peptide levels. We applied C-peptide to evaluate endogenous insulin levels. Fasting insulin sensitivity index was determined with the following equation: IS_HOMA-cp_ = 22.5/ (fasting glucose × fasting C-peptide) [19, 20]. Systemic insulin sensitivity index was calculated by the following equation: ISI_M-cp_ = 10,000/square root of (fasting glucose × fasting C-peptide) × (mean glucose × mean C-peptide during OGTT) [19, 20]. The area under the C-peptide curve (AUC_cp_) and the area under the glucose curve (AUC_glu_) were computed using the trapezoidal rule. The ratio of total area under the C-peptide curve to area under the glucose curve (AUC_cp/glu_) was used to evaluate the insulin secretion index. ISSI2_cp_ was calculated by multiplying AUC_cp/glu_ and ISI_M-cp_ [21, 22].

### Statistical analyses

Statistical analyses of the data were performed using SPSS (version 25.0) software. All participants were categorized by quartiles of serum ADA levels. All continuous variables with normal distribution were expressed as the mean ± standard deviation (SD) or skewed distributions were expressed as median (interquartile range). Whereas categorical variables were expressed as frequencies (percentages). If the variables were non-normally distributed, logarithmic transformations were applied to achieve a normal distribution. The one-way analysis of variance (ANOVA) was performed to compare differences in the continuous variables between serum ADA levels quartiles, and the Chi squared test was performed to compare categorical variables among the four subgroups. Pearson’s correlation test and partial correlation test were conducted to analyze the correlation between serum ADA levels and ISSI2_cp_. Moreover, multiple linear regression analysis was performed to explore the relationship between serum ADA levels and ISSI2_cp_ as the dependent variable. A *p* value < 0.05 was considered to indicate a statistically significant.

## Results

### Clinical characteristics of study participants

A total of 1573 participants diagnosed with T2D were recruited in this study and divided into four subgroups according to serum ADA levels. The clinical characteristics of the participants according to the quartiles of serum ADA levels are shown in the Table 1. The recruited participants had normal serum ADA levels of 13.6 (11.1–16.9) U/L, diabetes duration of 78.64 ± 74.45 months, ages of 58.17 ± 13.12 years, BMI of 25.75 ± 3.58 kg/m^2^ and HbA1c levels of 9.02 ± 4.43%. The serum ADA levels of the total participants were 13.6 (11.1–16.9) U/L, and of the quartiles were Q1 (9.5 (8.5–10.3)U/L), Q2 (12.4 (12.4–13.1)U/L), Q3 (15.0 (14.3–16.0)U/L) and Q4 (19.7 (18.1–21.7)U/L), respectively. As serum ADA levels quartiles increased, age, ratio of female to male, diabetes duration, ratio of hypertension, SBP, ALT and AST significantly increased (all *p* values for trend < 0.05), while BMI, DBP, TG, TC, HDLC and LDLC showed no differences (all *p* values for trend > 0.05). As the serum ADA levels quartiles increased, serum HbA1c increased from 8.19 ± 2.02% to 8.91 ± 1.92% to 9.06 ± 1.98% to 9.89 ± 2.15% (*p* < 0.001). Additionally, comparisons of glucose-lowering therapies showed that the frequency of insulin treatments and glucosidase inhibitors use were increased, whereas intervention by lifestyle alone was decreased, as serum ADA levels quartiles increased (all *p* values for trend < 0.05).

**Table 1:** Clinical characteristics of the study participants according to the quartiles of serum ADA levels

Variables	Total	Q1	Q2	Q3	Q4	*p* value
ADA(U/L)	13.6 (11.1–16.9)	9.5 (8.5–10.3)	12.4 (12.4–13.1)	15.0 (14.3–16.0)	19.7 (18.1–21.7)	< 0.001
n	1573	396	399	388	390	–
Age (year)	58.17 ± 13.12	53.79 ± 11.98	56.74 ± 12.78	59.12 ± 12.94	63.13 ± 13.01	< 0.001
Female (F/M)	683/890	125/271	176/223	182/206	200/190	< 0.001
Diabetes duration (months)	78.64 ± 74.45	63.5	76.56 ± 71.82	81.01 ± 74.15	93.80 ± 85.09	< 0.001
Glucose-lowering therapies						
Lifestyle alone, n (%)	105	35 (33.3)	22 (21.0)	35 (24.7)	13 (12.4)	0.003
Insulin treatments, n (%)	805	158 (19.6)	203 (25.2)	203 (25.2)	241 (29.9)	< 0.001
Insulin-secretagogues, n (%)	822	199 (24.2)	219 (26.6)	191 (23.2)	213 (25.9)	0.257
Metformin, n (%)	904	236 (26.1)	233 (25.8)	215 (23.8)	220 (24.3)	0.632
Pioglitazone, n (%)	571	137 (24.0)	148 (25.9)	133 (23.3)	153 (26.8)	0.433
Glucosidase inhibitors, n (%)	388	66 (17.0)	91 (23.5)	103 (26.5)	128 (33.0)	< 0.001
DPP-4Is, n (%)	433	93 (21.5)	118 (27.3)	103 (23.8)	119 (27.5)	0.111
Statin medication, n (%)	567	128 (22.6)	161 (28.4)	140 (24.7)	138 (24.3)	0.129
Hypertension, n (%)	804 (51.1)	177 (44.7)	205 (51.4)	198 (51.0)	224 (57.4)	0.005
BMI (kg/m^2^)	25.75 ± 3.58	25.51 ± 3.47	25.82 ± 3.49	25.85 ± 3.76	25.82 ± 3.62	0.518
SBP (mmHg)	137.20 ± 18.02	134.86 ± 17.80	137.36 ± 17.75	137.78 ± 17.49	138.85 ± 18.86	0.016
DBP (mmHg)	79.81 ± 10.89	80.24 ± 10.51	80.09 ± 10.48	79.93 ± 11.24	78.96 ± 11.31	0.349
ALT (U/L)	18 (13–28)	17 (12–26)	19 (13–28)	19 (14–30)	19 (13–29)	0.023
AST (U/L)	17 (14–22)	15 (13–20)	16 (14–21)	17 (14–23)	18 (15–25)	< 0.001
TG (mmol/L)	1.83 (1.18–2.92)	1.75 (1.11–2.83)	1.99 (1.27–3.15)	1.75 (1.18–2.86)	1.83 (1.21–2.84)	0.138
TC (mmol/L)	4.51 ± 1.36	4.41 ± 1.06	4.59 ± 0.98	4.56 ± 1.44	4.50 ± 1.14	0.172
HDLC (mmol/L)	1.04 ± 0.27	1.05 ± 0.28	1.04 ± 0.25	1.04 ± 0.28	1.03 ± 0.28	0.757
LDLC (mmol/L)	2.57 ± 0.79	2.52 ± 0.82	2.64 ± 0.78	2.57 ± 0.76	2.54 ± 0.82	0.225
HbA1c (%)	9.02 ± 4.43	8.19 ± 2.02	8.91 ± 1.92	9.06 ± 1.98	9.89 ± 2.15	< 0.001

The islet beta cell function indexes derived from OGTT for the all participants and the four subgroups are summaried in Table 2. The serum glucose levels at 0, 30, 60, 120, and 180 min were significantly increased from Q1 to Q4 of serum ADA levels (all *p* values for trend < 0.05). The serum C-peptide levels at 30, 60 and 120 min were significantly decreased from Q1 to Q4 of serum ADA levels (all *p* values for trend < 0.05), whereas there were no differences of serum C-peptide levels at 0 and 180 min among the four subgroups (all *p* values for trend > 0.05). Furthermore, the major metabolic parameters IS_HOMA-cp_, ISI_M-cp_, AUC_cp/glu_ and ISSI2_cp_ were decreased from QI to Q4 of serum ADA levels (all *p* values for trend < 0.01).

**Table 2:** Islet β cell function indexes of the study participants according to the quartiles of serum ADA levels

Variables		Total	Q1		Q2	Q3	Q4	*p* value
ADA (U/L)	13.6 (11.1–16.9)			9.5 (8.5–10.3)	12.4 (12.4–13.1)	15.0 (14.3–16.0)	19.7 (18.1–21.7)	< 0.001
n	1573			396	399	388	390	–
Glu0 (mmol/L)	11.39 ± 3.54			10.05 ± 3.32	11.29 ± 3.24	11.43 ± 3.39	12.83 ± 3.66	< 0.001
Glu30 (mmol/L)	11.99 ± 2.75			11.71 ± 2.55	12.06 ± 2.68	12.05 ± 2.87	12.15 ± 2.88	0.024
Glu60 (mmol/L)	15.33 ± 3.34			15.02 ± 3.26	15.27 ± 3.22	15.37 ± 3.38	15.66 ± 3.47	0.007
Glu120 (mmol/L)	16.53 ± 4.11			15.30 ± 4.21	16.25 ± 3.90	16.90 ± 3.99	17.68 ± 3.98	< 0.001
Glu180 (mmol/L)	12.67 ± 4.23			11.06 ± 4.00	12.28 ± 4.11	13.18 ± 4.13	14.19 ± 4.05	< 0.001
CP0 (ng/mL)	1.17 (0.78–1.75)			1.26 (0.83–1.75)	1.15 (0.74–1.75)	1.18 (0.74–1.82)	1.11 (0.80–1/69)	0.543
CP30 (ng/mL)	2.02 (1.36–2.89)			2.13 (1.49–3.12)	2.01 (1.34–2.90)	2.07 (1.33–2.87)	1.90 (1.25–2.70)	0.008
CP60 (ng/mL)	3.02 (2.04–4.38)			3.28 (2.35–4.64)	3.06 (2.07–4.46)	2.90 (2.05–4.38)	2.79 (1.83–3.86)	< 0.001
CP120 (ng/mL)	4.20 (2.94–5.98)			4.50 (3.16–6.26)	4.19 (2.83–6.02)	4.18 (2.93–5.99)	3.83 (2.71–5.55)	0.003
CP180 (ng/mL)	5.28 (2.72–5.28)			3.85 (2.74–5.36)	3.69 (2.65–5.10)	3.79 (2.79–5.39)	3.73 (2.65–5.25)	0.805
IS_HOMA-cp_	1.79 (1.20–2.52)			1.95 (1.37–2.72)	1.82 (1.24–2.58)	1.86 (1.16–2.56)	1.56 (1.06–2.33)	< 0.001
ISI_M-cp_	456.62 (328.50–619.82)			484.06 (355.65–646.62)	467.68 (331.12–620.50)	450.20 (320.57–622.24)	425.43 (306.25–563.45)	0.001
AUC_cp_	9.84 (6.93–13.70)			10.64 (7.47–14.31)	9.74 (6.84–13.96)	9.88 (6.92–14.01)	9.30 (6.42–12.73)	0.005
AUC_glu_	41.96 ± 9.06			39.48 ± 8.92	41.47 ± 8.48	42.67 ± 9.02	44.28 ± 9.15	< 0.001
AUC_cp/glu_	0.23 (0.15–0.35)			0.27 (0.18–0.39)	0.23 (0.15–0.34)	0.23 (0.14–0.35)	0.20 (0.14–0.30)	< 0.001
ISSI2_cp_	100.24 (70.43–142.99)			121.26 (84.84–178.36)	100.48 (73.23–138.89)	96.67 (69.63–140.94)	81.79 (60.18–116.89)	< 0.001

### Relationship between serum ADA levels and islet beta cell function

**Fig. 1:**
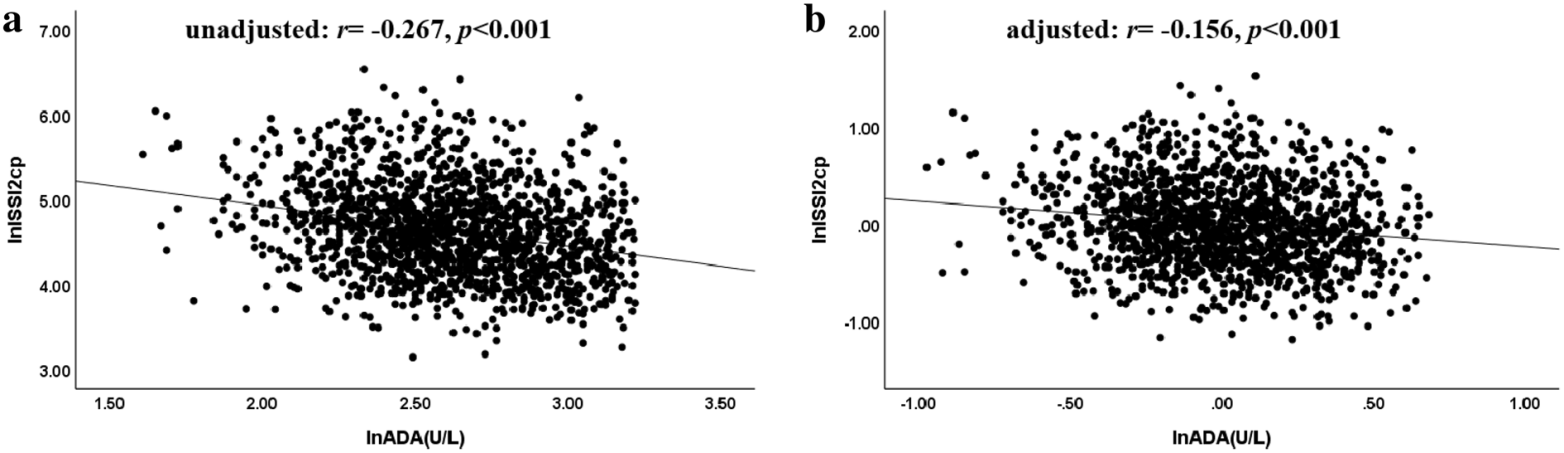
The relationship between the serum ADA levels and ISSI2_cp_ (**a** unadjusted;** b** adjusted for HbA1c)

The results of Person correlation between serum ADA levels and ISSI2_cp_ was presented in Fig. 1. The serum ADA levels were significantly and negatively correlated with ISSI2_cp_ (*r* = − 0.267, *p* < 0.001). Moreover, after adjusting for HbA1c, the association between serum ADA levels and ISSI2_cp_ was still existed (*r* = − 0.156, *p* < 0.001).

### Multiple liner regression analysis with islet beta cell function index as the dependent variable

The association of serum ADA levels and islet beta cell function index (ISSI2_cp_) was showed in Table 3 by multiple linear regression analyses, with ISSI2_cp_ as the dependent variable. In the basal unadjusted model 1, serum ADA levels were significantly associated with ISSI2_cp_ (*β* =  − 0.267, *t* =  − 10.994, *p* < 0.001, adjusted *R*^*2*^ = 0.071). In model 2, we adjusted for age, sex, BMI, SBP, DBP, diabetes duration, ALT, AST, TG, TC, HDLC, LDLC, Cr, IS_HOMA-cp_ and HbA1c and observed a gradual increase in the adjusted *R*^*2*^. In the fully adjusted model 3 (further adjusted for statin medication and glucose-lowering therapies), serum ADA levels were still independently associated with ISSI2_cp_ (*β* =  − 0.125, *t* =  − 5.397, *p* < 0.001, adjusted *R*^*2*^ = 0.459).

**Table 3:** Multiple liner regression analysis on ISSI2_cp_

Model	*β*	*t*	*p* value	Adjusted *R*^*2*^
Model 1	− 0.267	− 10.994	< 0.001	0.071
Model 2	− 0.131	− 5.539	< 0.001	0.426
Model 3	− 0.125	− 5.397	< 0.001	0.459

Model 1: Unadjusted

Model 2: Adjusted for age, sex, BMI, SBP, DBP, diabetes duration, ALT, AST, TG, TC, HDLC, LDLC, IS_HOMA-cp_ and HbA1c

Model 3: Additionally adjusted for statin medication and glucose-lowering therapies

## Discussion

In the current study, we analyzed the relationship between serum ADA levels in the normal range and insulin sensitivity, insulin secretion and islet beta cell function in patients with T2D. The main finding of the current study showed that serum ADA levels were inversely associated with islet beta cell function assessed by ISSI2_cp_.

The pathogenic mechanisms of T2D are insulin resistance and islet beta cell dysfunction, which characterized by chronic low-grade inflammation. Previous studies have proven that the defects in insulin action of T-lymphocyte may play an important role in inappropriate immune responses in T2D [24]. ADA is more active in T-lymphocyte than in B-lymphocyte,  and it plays an important role in lymphocyte proliferation, differentiation, and maturation [25, 26]. A small-scale study by Khemka et al. [17] demonstrate that serum ADA levels were significantly higher in nonobese T2D subjects than in healthy controls, and were positively correlation with FPG in these T2D subjects. A recent cross-sectional study found that serum ADA levels were significantly increased in uncontrolled diabetes (HbA1c > 7%) when compared to healthy controls and controlled diabetes (HbA1c < 7%), and were significantly positive correlated with FPG and HbA1c [5]. And our study showed that as serum ADA levels quartiles increased, FPG and HbA1c were increased. Our study is consistent with these previous findings. Thus,  it is suggested that the increased serum ADA levels may indicate the immune dysfunction and poor glycemic control in T2D.

Previous studies have shown that ADA is an important enzyme that modulates the insulin bioactivity [6, 27, 28]. In addition, ADA is an important enzyme in regulating adenosine concentration by inactivation of adenosine, and adenosine plays a critical role in modulating glucose and insulin homeostasis and the pathophysiology of T2D [7, 8, 29]. Dhalla et al. [30] revealed that adenosine A_1_ receptor agonist decreased the levels of free fatty acids (FFA) and triglycerides and improved the insulin resistance induced by high-fat diet in mice. Johansson et al. [31] demonstrated that adenosine increased insulin sensitivity for glucose transport in A_1_R knock out mice. Ohtani et al. [32] showed that the activation of adenosine A_2a_ receptor by adenosine resulted in increased insulin secretion in mouse pancreatic islets. C-peptide levels reflect endogenous insulin secretion more accurately than insulin levels. Our study showed that serum ADA levels were negatively correlated with fasting insulin sensitivity index measured by IS_HOMA-cp_, systemic insulin sensitivity index measured by ISI_M-cp_ and insulin secretion index measured by AUC_cp/glu_. Our data in a large population were consistent with previous studies. Therefore, serum ADA levels were negatively correlated with insulin sensitivity and insulin secretion in patients with T2D.

Serum ADA levels may also be a potential biomarker closely related to overall islet beta cell function. We applied ISSI2_cp_ to evaluate integrated islet beta cell function. Retnakaran et al. [20, 22] proposed that ISSI2_cp_ was a potential OGTT-based method for measuring islet cell function. And ISSI2_cp_ has been proven to measure the ability of islet beta cell to compensate for changes in systemic insulin sensitivity through changes in insulin secretion [33]. There are few studies about ADA and islet beta cell function, while a large number of studies have suggested that adenosine plays a role in regulating proliferation and survival of beta cell [32, 34]. Andersson et al. [35] demonstrated that adenosine agonist increased the proliferation of beta cell and accelerated the recovery of normoglycemia through the adenosine A_2aa_ receptor in  a zebrafish model of diabetes. Our study proved that serum ADA levels were negatively correlated with ISSI2_cp_. Moreover, our study suggested that serum ADA levels were independently associated with ISSI2_cp_ after multiple liner stepwise regression analysis in patients with T2D. Further research is needed to explore the relationship between ADA and islet beta cell function, and to explore the causal relationship among them.

There are few studies on the relationship between ADA and insulin sensitivity, insulin secretion and islet beta cell function. And their mechanism remains unclear, but their relationship may be explained as follows. Adenosine stimulates insulin activity in the processes of lipid synthesis, glucose transport, leucine oxidation and pyruvate dehydrogenase activity. Adenosine plays a critical role in regulating the activity of insulin in liver tissue, adipose tissue, cardiac muscle and skeletal muscle [8, 16, 36, 37]. Moreover, adenosine regulates the homeostasis of beta cell by controlling the proliferation and regeneration of beta cell [34, 38, 39]. ADA is a critical enzyme to regulate adenosine concentration. Thus, higher ADA activity in patients with T2D reduces adenosine levels which affects glucose homeostasis. Moreover, DDPIV/CD26 is a transmembrane glycoprotein. On the surface of T lymphocytes, ADA binds to DDPIV via adenosine A_2b_ receptor, which inhibits the glucagon-like peptide-1 (GLP-1) [16, 40]. And GLP-1 plays an important role in promoting insulin secretion, inhibiting glucagon secretion and stimulating the proliferation and differentiation of islet beta cell in patients with diabetes. In patients with T2D, the relationship between ADA and islet beta cell and its mechanism are still unclear, which needs the further study.

Several limitations of our study should be addressed. First, the current study was a cross-sectional observational study that cannot determine the causal association between serum ADA levels and islet beta cell function. Besides, Prospective longitudinal studies are needed to assess the casual relationship. Second, hyperglycemic and euglycemic clamp techniques are the gold standard for evaluating insulin secretion and insulin sensitivity, respectively. However, insulin sensitivity and islet beta cell function derived from the OGTT was more practical for the large-scale clinical study. Third, the current study may be restricted to Chinese population and lack generalizability to other population. Fourth, ADA has two isoenzymes ADA1 and ADA2, which may paly different roles in T2D and other metabolic disease. This may require further research to distinguish between the two isoenzymes for clinical relevance analysis. Fifth, after adjusting for other clinical confounders via multiple linear regression analyses, serum ADA levels were still independently associated with ISSI2_cp_. The *β* value is relative small (*β* = − 0.125), although it is significant. Therefore, we need more experiments to further investigate the relationship between serum ADA levels and islet beta cell function and their mechanism.

## Conclusions

In conclusion, serum ADA levels within the normal range are negatively associated with islet beta cell function assessed by ISSI2_cp_ in patients with T2D.

## Data Availability

The current data are available to all interested researchers upon reasonable request. Requests for access to data should be made to the principal investigators of the study.

## Abbreviations

ADA: Adenosine deaminase
T2D: Type 2 diabetes
FPG: Fasting plasma glucose
PPG: Postprandial plasma glucose
HbA1c: Glycated hemoglobin
OGTT: Oral glucose tolerance test
ISI_M-cp_: Matsuda index
AUC_cp__/__glu_: The ratio of total area under the C-peptide curve to area under the glucose curve
ISSI2_cp_: Insulin secretion-sensitivity index 2
SBP: Systolic blood pressure
DBP: Diastolic blood pressure
BMI: Body mass index
TG: Triglyceride
TC: Total cholesterol
HDLC: High-density lipoprotein cholesterol
LDLC: Low-density lipoprotein cholesterol
ALT: Alanine aminotransferase
AST: Aspartate aminotransferase
Cr: Creatinine
FFA: Free fatty acids
GLP-1: Glucagon-like peptide-1
